# Progesterone Attenuates Experimental Subarachnoid Hemorrhage-Induced Vasospasm by Upregulation of Endothelial Nitric Oxide Synthase via Akt Signaling Pathway

**DOI:** 10.1155/2014/207616

**Published:** 2014-05-13

**Authors:** Chia-Mao Chang, Yu-Feng Su, Chih-Zen Chang, Chia-Li Chung, Yee-Jean Tsai, Joon-Khim Loh, Chih-Lung Lin

**Affiliations:** ^1^Graduate Institute of Medicine, College of Medicine, Kaohsiung Medical University, 100 Shih-Chuan 1st Road, Kaohsiung 80708, Taiwan; ^2^Department of Neurosurgery, Kaohsiung Medical University Hospital, No. 100, Tzyou 1st Road, Kaohsiung 80708, Taiwan; ^3^Department of Surgery, Kaohsiung Municipal Hsiao Kang Hospital, No. 482, Shanming Road, Siaogang District, Kaohsiung 81267, Taiwan; ^4^Faculty of Medicine, Graduate Institute of Medicine, College of Medicine, Kaohsiung Medical University, 100 Shih-Chuan 1st Road, Kaohsiung 80708, Taiwan; ^5^Department of Surgery, Kaohsiung Municipal Ta-Tung Hospital, Kaohsiung No. 68, Jhonghua 3rd Road, Cianjin District, Kaohsiung City 80145, Taiwan

## Abstract

Cerebral vasospasm is the leading cause of mortality and morbidity in patients after aneurysmal subarachnoid hemorrhage (SAH). However, the mechanism and adequate treatment of vasospasm are still elusive. In the present study, we evaluate the effect and possible mechanism of progesterone on SAH-induced vasospasm in a two-hemorrhage rodent model of SAH. Progesterone (8 mg/kg) was subcutaneously injected in ovariectomized female Sprague-Dawley rats one hour after SAH induction. The degree of vasospasm was determined by averaging the cross-sectional areas of basilar artery 7 days after first SAH. Expressions of endothelial nitric oxide synthase (eNOS) and phosphorylated Akt (phospho-Akt) in basilar arteries were evaluated. Prior to perfusion fixation, there were no significant differences among the control and treated groups in physiological parameters recorded. Progesterone treatment significantly (*P* < 0.01) attenuated SAH-induced vasospasm. The SAH-induced suppression of eNOS protein and phospho-Akt were relieved by progesterone treatment. This result further confirmed that progesterone is effective in preventing SAH-induced vasospasm. The beneficial effect of progesterone might be in part related to upregulation of expression of eNOS via Akt signaling pathway after SAH. Progesterone holds therapeutic promise in the treatment of cerebral vasospasm following SAH.

## 1. Introduction

Aneurysmal subarachnoid hemorrhage (SAH) is a serious and fatal disease. The mortality rate is 27% to 44% in SAH patients [1], and 46% of SAH patients survive with serious sequela of cognitive and functional impairment [2]. The main therapy against SAH is securing the cerebral aneurysms and treating the cerebral vasospasm, which develops in 70% of SAH populations between 3 and 14 days after SAH onset [3]. Cerebral vasospasm causes delayed cerebral ischemia and contributes to the major cause of poor outcome and even death in SAH patients [1], but so far there is no definitive treatment against the devastating complication.

Growing evidence shows that progesterone, a sex steroid hormone, attenuates brain edema [4] and has beneficial effects on traumatic brain injury [5, 6], stroke [7, 8], experimental autoimmune encephalomyelitis [9, 10], and experimental spinal cord injury [11].

Our previous studies showed that 17*β*-estradiol (E2) attenuates SAH-induced vasospasm by the prevention of augmentation of inducible nitric oxide synthase (iNOS) expression and the preservation of normal eNOS expression that reduces secondary brain injury after SAH [12, 13]. The prevention of increased iNOS expression is achieved by interfering with the nuclear factor kappa B transactivation via estrogen receptor-dependent mechanism. Our other findings have also demonstrated that E2 reverses the decreased expression of adenosine A1 receptors and increases the expression of adenosine A2A receptors to prevent vasospasm and apoptosis induced by SAH in the dentate gyrus [14]. E2 treatment may reverse apoptosis by increasing phospho-Akt, ERK, and ER*α* protein expression in the dentate gyrus via an ER*α*-dependent pathway [15, 16]. Likewise, progesterone, a sex hormone like E2, has the potential role in treating SAH-induced vasospasm.

Endothelial nitric oxide (NO), enhanced by eNOS, is essential to vascular homeostasis and angiogenesis [17]. And eNOS is activated by various stimulations including the phosphatidylinositol 3-kinase- (PI3K-) protein kinase B/Akt signaling pathway, and phosphorylation of Akt is essential for its activity [18].

In this study, we hypothesized that reversal of spasm of basilar artery after SAH is due to activation of phospho-Akt, which subsequently increases eNOS expression after progesterone treatment. We used two-hemorrhage rodent model of SAH, measured the diameter of basilar artery, and examined the expression of eNOS and phospho-Akt in the basilar artery following SAH.

## 2. Materials and Methods

### 2.1. Animals

All experimental protocols were approved by the Kaohsiung Medical University Animal Research Committee. Ovariectomized female Sprague-Dawley rats (NLAC, Education Research Resource, National Laboratory Animal Center, Taiwan), weighing 300–350 gm, were used. The animals were maintained on a 12-hour light/dark cycle, with free access to food and water. Rats were evenly divided into the following four groups. Animals in group 1 served as controls and were not subjected to SAH (control; *n* = 20). The animals in all other groups were subjected to experimental SAH as described below. Group 2 received experimental SAH without additional treatment (SAH only; *n* = 20). Group 3 received experimental SAH plus vehicle (SAH + vehicle; *n* = 20). Group 4 received experimental SAH with progesterone (8 mg/kg/day, subcutaneously) treatment 1 hour after the first induction of SAH (SAH + P; *n* = 20) for 7 days following the first hemorrhage.

### 2.2. Induction of Experimental SAH

Rats were anesthetized by an intraperitoneal injection of pentobarbital (50 mg/kg). Animal's head was fixed in stereotactic frame and the cistern magna was punctured percutaneously with a 25-gauge butterfly needle. About 0.1 to 0.15 mL of CSF was slowly withdrawn and the junction of butterfly needle and tube was clamped. Freshly autologous nonheparinized blood (0.3 mL) was withdrawn from tail artery. Using a needle-in-needle method (inserting 30-gauze needle into 25-gauze butterfly needle at the junction of needle and tube), blood was injected slowly into the cistern magna in approximately 2 minutes. The same procedure was repeated 48 hours later. Seven days after the first SAH, animals were sacrificed by perfusion and fixation. Then the brain was removed, placed in a fixative solution, and stored at 4°C overnight. The basilar arteries were isolated for further examination.

### 2.3. Basilar Artery Morphometric Analysis

Morphometric measurements were performed by an investigator blinded to the treatment groups. At least five random arterial cross-sections from each animal were evaluated qualitatively for the extent of corrugation of the internal elastic lamina (IEL), and the cross-sectional area of each section was measured using a computer-assisted image analysis system. The areas of the five cross-sections from basilar artery were averaged to provide a single value for each animal. Group data were expressed as mean ± SEM. Group comparisons were performed using a one-way analysis of variance (ANOVA). Differences were considered significant at the *P* < 0.05 level.

### 2.4. Western Blot Analysis

Six animals in each group were included in this protocol. Samples were obtained from the basilar artery (BA). BA tissue was homogenized in ice-cold M-PER Mammalian Protein Extraction Reagent (Pierce, Rockford, IL) with Protease inhibitor (Complete Mini; Roche, Mannheim, Germany), centrifuged at 15000 rpm for 20 minutes. The protein concentration was estimated using the Bio-Rad protein microassay procedure. Samples were heated for 5 minutes in boiling water. Equal amounts of protein were loaded in each land of SDS-PAGE. The gels were transferred onto polyvinylidene difluoride (PVDF; PerkinElmer, Waltham, MA) membrane by electroblotting for 90 minutes (100 V), and the membrane was blocked overnight at 4°C with the Tween-Tris buffer saline solution (T-TBS; 20 mM Tris base, 0.44 mM NaCl, 0.1% Tween 20, and pH 7.6) containing 5% nonfat dry milk and 0.1% Tween 20. The blot was incubated with primary antibodies eNOS (1 : 1000; BD), phospho-Akt (1 : 1000; Cell Signaling Technology, Inc., Beverly, MA), Akt (1 : 4000; Cell Signaling Technology, Inc.), and *β*-actin (1 : 40000; Sigma, St. Louis, MO) and then rinsed with T-TBS for 30 minutes and incubated with goat anti-mouse IgG antibody conjugated to horseradish peroxidase (Jackson ImmunoResearch, West Grove, PA). Akt and *β*-actin levels served as an internal standard and to account for loading differences. Membranes were rinsed with T-TBS for 30 minutes, incubated with electrochemiluminescence reagent (PerkinElmer, Waltham, Massachusetts) for 2 minutes, and apposed to the manufacturer's specification. We scanned the X-ray films and the determined optical density by employing ImageJ (National Institutes of Health, Bethesda, MD).

### 2.5. Statistics

The data were expressed as mean ± standard error of the mean. Differences between the experimental groups were determined with one-way analysis of variance with the Bonferroni post hoc test. Differences were accepted as significant at the *P* < 0.05 level.

## 3. Results

### 3.1. General Observations

Prior to perfusion fixation, there were no significant differences among each group of rats in physiological parameters, including body weight, mean arterial blood pressure, and heart rate (data not shown).

### 3.2. Basilar Artery Cross-Sectional Luminal Area Measurements

**Figure 1:**
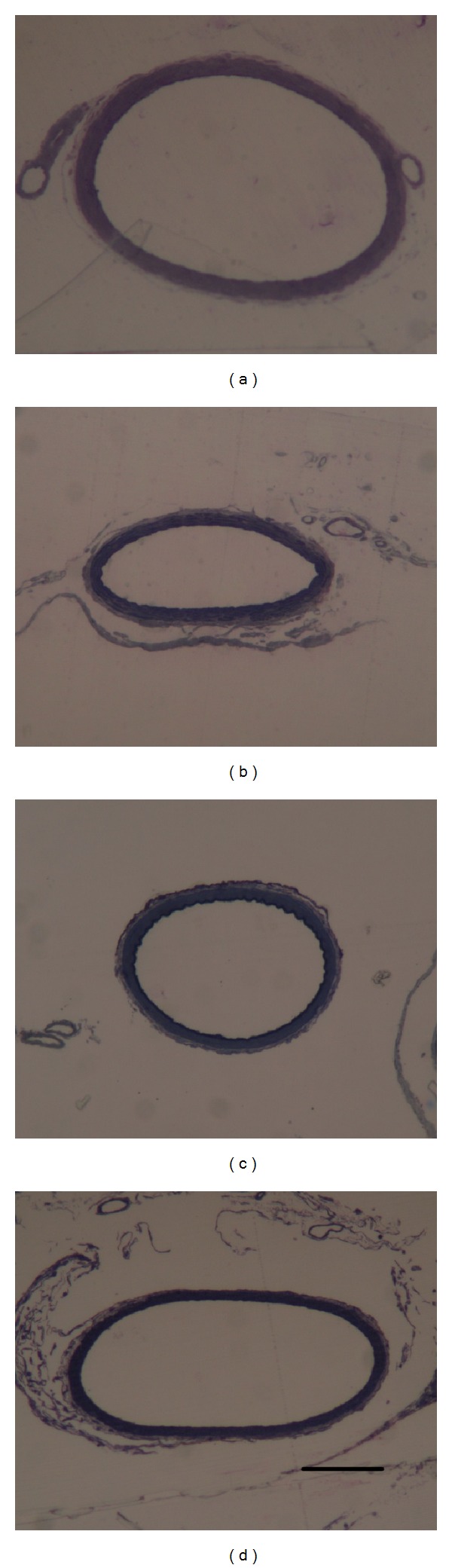
Photomicrographs of representative cross-sections of basilar arteries from control (a), SAH only (b), SAH plus vehicle treatment (c), and SAH plus progesterone treatment (d) (scale bar = 80 *μ*m).

**Figure 2:**
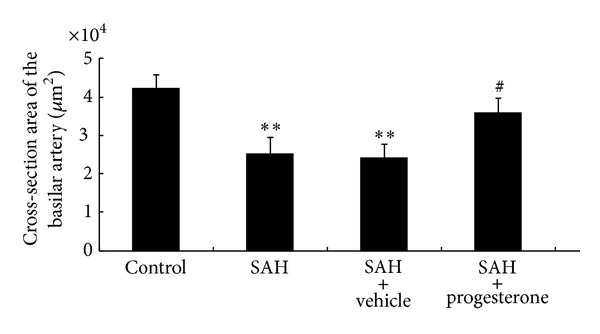
Bar graphs showing the effects of progesterone on cerebral vasospasm in cross-sectional areas. The average luminal area (mean ± SEM) of basilar arteries is demonstrated for each group of animals. Conversely, the basilar artery luminal areas of the progesterone treated group were markedly larger than those in the SAH-only group. ^#^ *P* < 0.05 compared with the SAH-only group; **P < 0.01, significantly different from the control group.

The cross-sectional area of basilar arteries was significantly reduced in animals subjected to SAH. Compared with the control group (42255.8 ± 3563.7 *μ*m^2^), the areas in the SAH only (25121.5 ± 4361.9 *μ*m^2^) and SAH plus vehicle groups (24020.3 ± 3716.1 *μ*m^2^) were reduced by 41% (*P* < 0.01) and 43% (*P* < 0.01), respectively (Figures 1 and 2). The cross-sectional areas in the progesterone treatment group (35802.3 ± 3960.5 *μ*m^2^) differed significantly from those of the SAH-only group (*P* < 0.01). There was no significant difference between the progesterone treatment group and the control group.

### 3.3. The Expression of eNOS and Phospho-Akt Proteins in Basilar Artery

**Figure 3:**
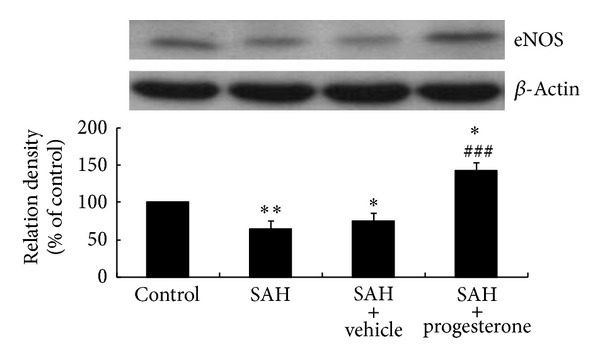
Bar graphs showing progesterone treatment inducing the increase in eNOS protein expression within the basilar artery following SAH. ^#^ *P* < 0.05, significantly different from the SAH group; ^###^ *P* < 0.001, significantly different from the SAH group; *P < 0.05, significantly different from the control group; **P < 0.01, significantly different from the control group.

The eNOS protein content decreased significantly in the SAH group (*P* < 0.01) and SAH plus vehicle group (*P* < 0.05) when compared with the control group. The eNOS protein content increased significantly in the progesterone treatment group when compared with SAH group (*P* < 0.001) and control group (*P* < 0.05) (Figure 3).

**Figure 4:**
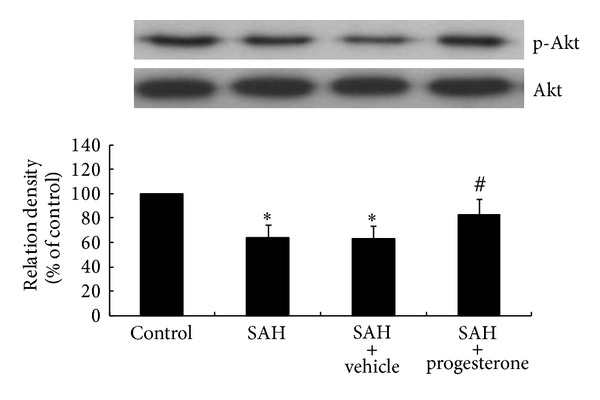
Bar graphs showing progesterone treatment enhancing phospho-Akt protein expression within the basilar artery following SAH. ^#^ *P* < 0.05, significantly different from the SAH group; *P < 0.05, significantly different from the control group.

The phospho-Akt protein content decreased significantly in the SAH group (*P* < 0.05) and SAH plus vehicle group (*P* < 0.05) when compared with the control group. The phospho-Akt protein content increased significantly in the progesterone treatment group when compared with the SAH group (*P* < 0.05). No significant difference of phospho-Akt protein amount was observed between the progesterone treatment group and control group (Figure 4).

## 4. Discussion

Treatments for cerebral aneurysms and associated vasospasm are complicated. Besides, patients with vasospasm have more inpatient costs and longer hospital stays [19]. Presently, no definite medical treatment is effective against vasospasm. Clinically we use oral nimodipine for patients with SAH, but nimodipine improves functional outcome not contributing to the improvement of vasospasm but contributing to its neuroprotective effect [20]. Recently, a phase 3 randomized trial, MASH-2, published its results that magnesium sulfate does not improve clinical outcome in 1,203 patients with aneurysmal SAH [21]. Progesterone has been proven to show beneficial effects on traumatic brain injury and stroke in experimental models. O'Connor and colleagues showed that progesterone decreased the apoptosis in hippocampus and cortex and improved the motor performance after diffuse traumatic brain injury in rats [5]. Wang and colleagues demonstrated that progesterone reduced the infraction volume, decreased brain edema, and improved functional outcome in middle cerebral artery occlusion rats [7]. Our study showed that progesterone has therapeutic effects in SAH-induced vasospasm.

Currently, few studies have investigated the role of progesterone in SAH or SAH-induced vasospasm. One study from Yan et al. showed that progesterone decreased brain water content, restored blood-brain barrier, and decreased expression of MMP-9 and caspase-3 in rats within 24 hours after SAH onset [22], which demonstrated that progesterone had neuroprotective effects mainly in early brain injury after SAH. To our knowledge, our study is the first study to apply progesterone in the treatment of SAH-induced vasospasm, which causes delayed brain injury.

In the present study, the expression of eNOS decreased markedly after SAH and treatment with progesterone enhanced the expression of eNOS and reversed the vasospasm of basilar arteries. Khurana et al. demonstrated that expression of recombinant eNOS in the BA relieved SAH-induced vasospasm [23]. In our laboratory, increased expression of eNOS mRNA was also proven to prevent SAH-induced vasospasm via treatment of E2 in experimental models [12]. In addition to SAH-induced vasospasm, NO produced by eNOS elicits vasodilation and consequent neuroprotective effects after brain ischemia [24]. NO is a potent vasodilator and in the condition of vasospasm, any treatment related to eNOS upregulation and NO production would be a promising therapy. In addition to cerebral arteries, progesterone was also shown to increase NO production by upregulating expression of eNOS in endothelial cells of rat aortas [25] and human umbilical veins [26]. Accordingly, progesterone has an important role in activating eNOS in vascular endothelial cells.

NO is essential for vascular tone regulation and it is induced by eNOS, which is stimulated by blood shear stress at endothelial cells [27]. And Dimmeler et al. demonstrated that Akt phosphorylated the Serine 1177 site of eNOS protein and that enhanced eNOS activity; they inhibited the PI3K/Akt pathway and that led to prevention of eNOS activation [28], and Fulton et al. had similar findings [29]. These findings showed that Akt plays a crucial role in eNOS activity and this molecular mechanism is important in many ways, including endothelial cell migration and angiogenesis [30], E2-induced vasodilation [31], protection in ventilator-associated lung injury [32], and protection in intestinal tissue in the situation of intestinal ischemia by Akt-dependent activation of endothelial nitric oxide synthase and vasodilation [33]. Our previous studies showed that E2 attenuates SAH-induced vasospasm by the preservation of normal eNOS expression and reduces secondary brain injury by increasing phospho-Akt, ERK, and ER*α* protein expression in the dentate gyrus after SAH [12, 13, 15, 16]. And in this study, we suggest that progesterone also showed its antivasospasm effect in this pathway.

It has been shown that progesterone elicited neuroprotective effects in the brain via phosphorylation of Akt [34]. Furthermore, progesterone was also proven to decrease apoptosis via PI3K/Akt pathway in ischemic brain injury [8]. In addition, similar to our results, Khorram and Han demonstrated that progesterone stimulated the production of eNOS protein in human endometrial-derived epithelial cells, and the effect was inhibited completely by an inhibitor of PI3K/Akt pathway called wortmannin [35]. So these results strongly suggest that the effect of progesterone on eNOS was mediated via PI3K/Akt pathway.

## 5. Conclusion

Treatment with progesterone is effective in the prevention of vasospasm in a two-hemorrhage rodent model of SAH. The antivasospasm effect of progesterone may arise from increased expression of eNOS via the PI3K/Akt pathway after SAH. The use of progesterone is promising in the treatment of cerebral vasospasm following aneurysmal SAH and needs further investigation.
